# Use of Expert Relevancy Ratings to Validate Task-Specific Search Strategies for Electronic Medical Records

**DOI:** 10.2196/medinform.3205

**Published:** 2014-03-11

**Authors:** Harlan Harvey, Arun Krishnaraj, Tarik K Alkasab

**Affiliations:** ^1^Department of RadiologyMassachusetts General HospitalBoston, MAUnited States; ^2^Department of RadiologyUniversity of VirginiaCharlottesville, VAUnited States

**Keywords:** medical informatics, medical records systems, computerized, health information management

## Abstract

As electronic medical records (EMRs) grow in size and complexity, there is increasing need for automated EMR tools that highlight the medical record items most germane to a practitioner’s task-specific needs. The development of such tools would be aided by gold standards of information relevance for a series of different clinical scenarios. We have previously proposed a process in which exemplar medical record data are extracted from actual patients’ EMRs, anonymized, and presented to clinical experts, who then score each medical record item for its relevance to a specific clinical scenario. In this paper, we present how that body of expert relevancy data can be used to create a test framework to validate new EMR search strategies.

## Introduction

### Electronic Medical Records

As electronic medical records (EMR) become more common throughout the medical community, a wider variety of structured and unstructured data are being incorporated into them. Increasing EMR content has meant that some data necessary for clinical decision making are spread among several documents and repositories. This has the potential to increase practitioner workload, predispose to medical errors, and result in unnecessary utilization of health care resources [1,2]. In an attempt to reclaim efficiency, practitioners may lean on unreliable heuristics to obtain the answers they need.

### Task-Specific Algorithms

Task-specific EMR search algorithms could ameliorate this situation by better addressing the diverse needs of practitioners [3-5]. However, one challenge in designing task-specific algorithms is finding a way to validate proposed search strategies prior to clinical implementation, given a lack of task-specific gold standards [6]. We have previously described a process for collecting context-specific expert relevancy ratings of medical record items [7]. The process relies on anonymized items of medical record data extracted from actual patients’ EMRs, which are then presented to and rated by clinical experts based on the medical record items relevancy to a specific clinical task or scenario. The resultant relevancy data collected by the process can serve as the gold standard against which to evaluate search algorithms.

In this paper, we describe how the expert relevancy ratings data can be employed as a test framework to validate search strategies. We include proposed formats for transmitting data between separate steps and a preliminary algorithm for assessing the concordance between the “hits” from a search strategy and the expert relevance ratings.

## Methods

### The Three Main Subprocesses

**Figure 1:**
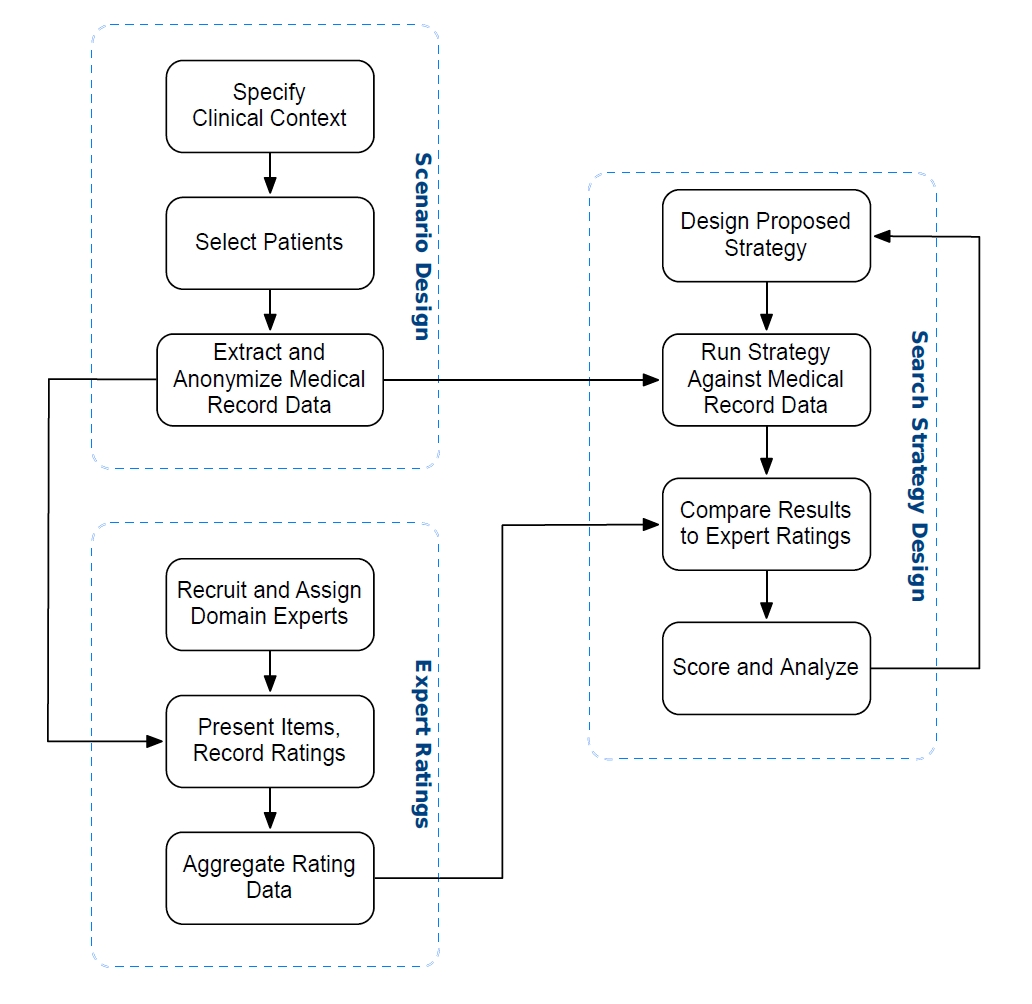
The flow of data through a process of validated medical record searches for a specific clinical context. For a defined clinical context, a set of representative patients is selected and medical record items are extracted and anonymized. These datasets are then presented to a panel of domain experts who generate a set of rating data. Meanwhile, an automated search to highlight relevant items is designed and then run against all of the anonymized medical record data to determine which items would be considered “hits.” This result set is then compared with the expert relevance ratings and a normalized score is generated which quantifies the level of agreement between the search and the experts, which can then be used to design improvements in the search.

There are three main subprocesses that are required to implement this vision for any given clinical scenario (Figure 1 shows these subprocesses). First, representative medical record data must be extracted from the EMR to serve as a target dataset. This dataset should incorporate a range of medical record items, chosen broadly from all items that might be available to a practitioner in the given clinical scenario. Second, this set of medical record items must be presented to a panel of clinical experts, who will rate each item for relevance in the given clinical scenario. Typically, these experts are clinical physicians who have been recruited because they frequently encounter the given clinical situation; their collected relevance ratings serve as a gold standard for the relevance of medical record items, which might or might not be included as a search result. Finally, a proposed search strategy will be generated and tested against the set of medical record items. The agreement between the items highlighted by the search algorithm and those rated as relevant by the experts can be computed and used as a performance metric for the search algorithm.

### Example Medical Record Data

For a given clinical scenario, a set of matching patients can be selected. A sample of matching medical record items can then be extracted from the EMR system and anonymized. This set of medical record items for one patient is deemed a scenario, and can be expressed as an eXtensible Markup Language (XML) data file matching the following RELAX NG Compact open source schema found at the referenced link [8,9]. This defines a <clinical_scenario> as including patient demographic information and data regarding an index examination. Then, a list of <medical_record_item>s is listed, along with information about the type of record and the number of days between the record and the index exam.

A set of such scenarios that are examples of a single clinical scenario is termed a <scenario_family>, as defined by the open source schema found at the referenced link [10]. A <scenario_family> contains numerous <scenario_reference>s. This definition, together with the relevant defined scenarios, forms the dataset against which a search strategy can be run.

### Expert Rating Data

Once a set of medical record data is available, it can be presented to a group of experts. The expert panel is made up of clinicians from the particular medical specialty tasked with the clinical scenario of interest. For example, if the method were being employed to identify medical record items pertinent to the clinical task of interpreting an MRI examination of the liver, the expert panel would be made up of abdominal radiologists knowledgeable in the clinical information germane to that task. The experts will rate each item for its relevance to the particular scenario along a four-step scale. The steps are labeled-“Irrelevant,” “Unlikely relevant,” “Probably relevant,” and “Certainly relevant.” These rating data can be gathered into an XML file that matches the open source schema, found at the referenced link [11]. The data are stored as a hierarchy of <scenario_family_ratings>, <scenario_ratings>, <rater_data>, and individual <item_rating>s.

### Search Strategy Results

The results of running a given search strategy against the medical record items contained in a <scenario_family> can be represented using an open source schema found at the referenced link [12]. The schema organizes a series of <item_result> elements, each of which indicates whether the given search strategy would include the given item as a hit or not.

### Strategy Scoring Metric

A scoring metric was developed for describing the extent of agreement between results returned by a particular search strategy and the expert rating data. The strategy is based on calculation of the kappa statistic [13]. This measure will be highest when experts agree on the relevance of an item of medical record data and the search strategy appropriately includes or excludes the item. Specifically, the statistic will increase monotonically with increasing agreement between a tested search strategy and the expert raters. After expert rating data have been collected for a given test set of medical record items and a candidate search strategy is tested against that same test set, these metrics are calculated to assess the performance of the candidate strategy.

## Results

**Figure 2:**
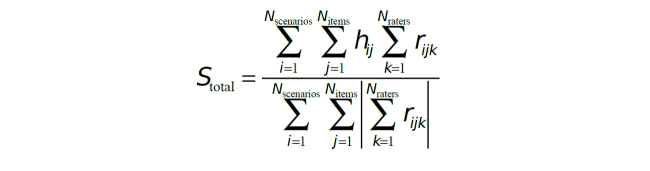
Equation to calculate a performance score for a search strategy based off of the expert relevancy ratings.

The overall performance of the search strategy is captured by a single metric, *S*_*total*_. Figure 2 shows this equation, where *N*_*scenarios*_, *N*_*items*_, and *N*_*raters*_ are the numbers of scenarios, medical record items, and raters. *h*_*ij*_ is +1 if the search strategy would include the *j* ^*th*^ item of the *i* ^*th*^ scenario as a hit, and -1 if it would not be included. *r*_*ijk*_ depends on the *k* ^*th*^ expert’s relevance rating of the *j* ^*th*^ item of the *i* ^*th*^ scenario–“Certainly relevant” is scored as +1, “Probably relevant” is scored as +½, “Unlikely relevant” is scored as -½, and “Irrelevant” is scored as -1. *S*_*total*_ is normalized to range from -1 (indicating perfect disagreement between the search results and the expert relevancy ratings) through 0 (indicating no correspondence between the search results and the expert relevancy ratings), and +1 (indicating perfect agreement between the search results and the expert relevancy ratings).

A metric for the degree of concordance only for relevant included items, *S*_*included*_, can also be calculated. This includes only items where ∑*r*_*ijk*_>0 (that is, items rated as overall relevant). This metric indicates the extent to which search results include relevant items (ie, the “sensitivity” of the search algorithm). The opposite metric, *S*_*excluded*_, which includes only items where ∑*r*_*ijk*_<0, indicates the degree to which items rated as irrelevant are excluded from the search algorithm results. Both of these metrics also range from -1 to +1.

These metrics can be represented according to the open source schema, available at the referenced link [14].

## Discussion

### Implications

Federal subsidies in the Health Information Technology for Economic and Clinical Health Act have essentially ensured that EMR will become commonplace in US health care facilities [15]. While capturing and presenting medical information in an electronic format is an important first step, the next steps in making this information useful will include the capability to perform a simple keyword search, followed by the development of more complex, context-specific searches [16,17]. Designing context-specific searches that are both accurate and complete is particularly challenging given the large amount of unstructured free text data within the medical record [4,16]. For instance, free text patient data may be characterized by abbreviations, synonyms, acronyms, negative forms of key terms, or misspellings, all of which must be incorporated into optimized search strategies [4]. Because of these recognized pitfalls in EMR search algorithm design, it is essential to be able to quantitatively judge and refine search algorithms in a cyclic iterative development pattern.

The process described herein would allow for the use of an interactive search strategy design tool. After loading the sample medical record data and relevance ratings, the designer could modify a search strategy’s metadata conditions and regular expressions and assess the overall performance changes. The tool could also be engineered to allow the designer to drill into the result set to find exemplars of the items that result in a mismatch of relevance ratings and search results. When an optimized search strategy is found, it is essentially prevalidated.

### Process Advantages

One advantage to the process outlined above is that by basing the sample data on real patient medical records and physicians’ specific impressions of which items are useful in a particular context, a very specific, detailed model of relevance is created which simple search heuristics are unlikely to capture well. As search strategy developers add complexity to their tools, they will be able to tell whether modifications are actually resulting in better matching.

The datasets and relevance tools can be shared, and even made semipublicly available. The universally unique identifiers attached to the scenario families, scenarios, medical record items, and raters minimize the chances of duplicated data. Individual sites can add their own patient data to already specified clinical scenarios and recheck performance given their site-specific sample data. Adding new raters and incorporating their responses can reduce the effect of individual raters’ idiosyncrasies. The library of clinical scenarios can be expanded over time and shared.

The initial conception of the tool was to aid radiologists who desire relevant medical record information at the time of interpretation. However, many medical practitioners would benefit from having relevant items in the medical record highlighted for them, especially if the tool’s accuracy for including relevance and excluding irrelevance is high. Additionally, these context-specific search strategies represent potentially powerful research tools, specifically related to outcomes tracking [6].

### Process Limitations

There are many limitations to the search strategy validation process as described. First, the process of collecting the expert relevancy ratings is only semiautomated and therefore time intensive. Collection of the data requires clinical personnel, many of whom are already stretched thin and working in an atmosphere of shrinking margins, to take time away from clinical duties to perform the relevancy rating. The long-term viability of this semiautomated process requires further study and continuous process improvements to reduce the impact on experts. Second, the process relies completely on relevancy ratings communicated using a nondichotomous, ordinal scale of values. As a result, the method of data collection and subsequent validation framework fails to capture potentially valuable qualitative feedback from expert raters. Potential future work can be aimed to provide further nuance to the validation framework by incorporating qualitative feedback, such as free text entries from expert raters. Last, since this work only proposes and lays out this process, future work will be needed to validate the method of calculating the performance score and to determine whether search strategies validated by the process are actually deemed as useful by clinical providers in their daily practice. We expect that this mode of calculated search strategy performance will be only one component of evaluating and improving search strategies. Other important metrics of performance as well as the subjective experience of the returned results should also be considered to evaluate automated search strategies deployed for clinical use.

In this paper, we have outlined a process for developing and validating context-specific search strategies based on context-specific expert relevancy ratings. Since both the method for collecting the expert relevancy ratings and the framework for validating search strategies are provided as open-source tools with open formats for data interchange, any research group or commercial entity can develop software to bring data into this process and perform the proposed steps. We anticipate that the formats and process will be further refined over time as it is adapted to new tasks and clinical applications.

## Abbreviations

EMR: electronic medical record
XML: eXtensible Markup Language
